# Thymoquinone-Loaded Chitosan Nanoparticles Combat Testicular Aging and Oxidative Stress Through SIRT1/FOXO3a Activation: An In Vivo and In Vitro Study

**DOI:** 10.3390/pharmaceutics17020210

**Published:** 2025-02-06

**Authors:** Enas A. Kasem, Gehan Hamza, Nagi M. El-Shafai, Nora F. Ghanem, Shawky Mahmoud, Samy M. Sayed, Mohammed Ali Alshehri, Laila A. Al-Shuraym, Heba I. Ghamry, Magdy E. Mahfouz, Mustafa Shukry

**Affiliations:** 1 Faculty of Science, Zoology Department, Kafrelsheikh University, Kafrelsheikh 33516, Egypt; 2 Nanotechnology Center, Chemistry Department, Faculty of Science, Kafrelsheikh University, Kafrelsheikh 33516, Egypt; 3 Department of Physiology, Faculty of Veterinary Medicine, Kafrelsheikh University, Kafrelsheikh 33516, Egypt; 4 Department of Economic Entomology and Pesticides, Faculty of Agriculture, Cairo University, Giza 12613, Egypt; 5 Department of Biology, Faculty of Science, University of Tabuk, Tabuk 71491, Saudi Arabia; ma.alshehri@ut.edu.sa; 6 Department of Biology, College of Science, Princess Nourah bint Abdulrahman University, P.O. Box 84428, Riyadh 11671, Saudi Arabia; laalshuraym@pnu.edu.sa; 7 Nutrition and Food Science, Department of Biology, College of Science, King Khalid University, P.O. Box 960, Abha 61421, Saudi Arabia; hebaghamry882011@gmail.com

**Keywords:** thymoquinone, chitosan nanoparticles, D-gal, oxidative stress, testicular protection

## Abstract

**Background:** Aging is a complex biological process characterized by the accumulation of molecular and cellular damage over time, often driven by oxidative stress. This oxidative stress is particularly detrimental to the testes, where it causes degeneration, reduced testosterone levels, and compromised fertility. D-galactose (D-gal) is commonly used to model aging as it induces oxidative stress, mimicking age-related cellular and molecular damage. Testicular aging is of significant concern due to its implications for reproductive health and hormonal balance. This research examines the protection by thymoquinone (TQ) or thymoquinone-loaded chitosan nanoparticles (NCPs) against D-galactose (D-gal)-induced aging in rat testes, focusing on biochemical, histological, and molecular changes. Aging, which is driven largely by oxidative stress, leads to significant testicular degeneration, reducing fertility. D-gal is widely used to model aging due to its ability to induce oxidative stress and mimic age-related damage. TQ, a bioactive ingredient of *Nigella sativa*, has earned a reputation for its anti-inflammatory, anti-apoptotic, and antioxidant characteristics, but its therapeutic application is limited by its poor bioavailability. **Methods**: Thymoquinone was loaded into chitosan nanoparticles (NCPs) to enhance its efficacy, and this was hypothesized to improve its stability and bioavailability. Four groups of male Wistar rats participated in the study: one for the control, one for D-gal, one for D-gal + TQ, and the last one for D-gal + NCP. **Results**: The results exhibited that D-gal substantially increased oxidative injury, reduced testosterone levels, and caused testicular damage. Treatment with TQ and NCPs significantly reduced oxidative stress, improved antioxidant enzyme levels, and restored testosterone levels, with NCPs showing a stronger protective effect than TQ alone. A histological analysis confirmed that NCPs better preserved testicular structure and function. Additionally, the NCP treatment upregulated the expression of key genes of oxidative stress resistance, mitochondrial function, and reproductive health, including SIRT1, FOXO3a, and TERT. **Conclusions:** The findings suggest that NCPs offer enhanced protection against aging-related testicular damage compared with TQ alone, which is likely due to the improved bioavailability and stability provided by the nanoparticle delivery system. This research emphasizes the potential of NCPs as a more effective therapeutic strategy for mitigating oxidative stress and age-related reproductive dysfunction. Future research should further explore the mechanisms underlying these protective effects.

## 1. Introduction

Aging eventually causes deterioration through regression in functional and physical capabilities, leading to oxidative stress, apoptosis, various illnesses, and, ultimately, mortality [1]. This process is largely driven by oxidative stress, which contributes to numerous diseases by accumulating reactive oxygen species (ROSs) and decreasing antioxidant defenses [2]. Among the organs affected by aging, the testis are particularly vulnerable, showing significant morphological and structural changes, including a reduced germ cell count and volume, leading to decreased fertility [3].

The D-galactose (D-gal) animal model is regularly used to study aging and anti-aging medicines [4]. Chronic exposure to D-gal in mice accelerates aging by inducing oxidative injury and increasing lipid peroxidation, resulting in cognitive decline and motor impairment similar to those in natural aging [5]. Considering the significance of aging and how it affects different biological systems, studying protective factors against aging is crucial [6]. Natural substances with therapeutic potential, particularly those with antioxidant and anti-inflammatory properties, have garnered significant attention for treating age-related conditions, including reproductive issues [6]. *Nigella sativa* L., or black seed, is a medicinal herb known for its effectiveness in treating various diseases, including reproductive disorders [7]. Studies have shown that black seed and its bioactive component, thymoquinone (TQ), have beneficial effects on sperm parameters, sex hormones, and reproductive organs in infertile men [8]. TQ’s anti-inflammatory, anti-apoptotic, antioxidant, and fertility-boosting properties provide strong testicular protection [8]. Research has demonstrated that TQ improves semen quality and reproductive functions while mitigating testicular damage induced by oxidative stress and apoptosis [8].

However, TQ’s limited stability, low oral bioavailability, and poor water solubility pose challenges to its therapeutic application, often requiring high doses for effectiveness [9]. In aqueous environments, TQ demonstrates limited stability, particularly in neutral to slightly alkaline conditions, leading to rapid degradation and oxidation, which diminishes its bioactivity [10]. This instability is exacerbated by its sensitivity to light, especially ultraviolet (UV) exposure, necessitating storage in light-protective, opaque containers to prevent photodegradation [10]. TQ’s poor water solubility, further limits its therapeutic application. However, it exhibits higher solubility in organic solvents such as ethanol, dimethyl sulfoxide (DMSO), and methanol, and it is more soluble in lipid-based mediums due to its lipophilic nature [11]. This lipophilicity has driven research on lipid-based drug delivery systems as potential carriers to improve TQ’s absorption and distribution within the body [11]. Bioavailability remains a significant challenge for thymoquinone (TQ). When administered orally, TQ undergoes rapid metabolic breakdown throughout the gastrointestinal tract, resulting in a poor bioavailability [12]. To address these limitations, various formulation strategies have been explored to enhance its stability and absorption. Encapsulation in liposomes or nanoemulsions has proven effective, as these systems can shield TQ from gastrointestinal degradation and improve its absorption [13]. For example, a self-nanoemulsifying drug delivery system (SNEDDS) was developed to increase TQ’s oral bioavailability, achieving a fourfold improvement compared with pure TQ [14]. Additionally, stable liposomal drug delivery systems have been investigated to further improve TQ’s solubility and bioavailability, ultimately leading to better therapeutic outcomes [15]. These formulation approaches offer promising solutions to overcome TQ’s inherent limitations, unlocking its full therapeutic potential.

In recent years, various formulations have been developed to augment the bioavailability, stability, and therapeutic efficacy of thymoquinone (TQ) in treating oxidative-stress-related disorders. Notably, TQ-loaded nanoparticles, including chitosan- and lipid-based nanoparticles, have gained attention due to their ability to improve the delivery and stability of TQ in biological systems [16]. This nanoparticle-based approach mirrors objectives seen in other TQ studies focused on oxidative damage and inflammation, as nanoparticles not only protect TQ from rapid degradation but also facilitate targeted delivery, increasing cellular uptake and therapeutic impact [17]. Similar strategies have been applied to other bioactive compounds where nano-formulations, such as liposomes or polymeric nanoparticles, enhance drug solubility, stability, and bioavailability, further supporting the promise of nanoparticle systems as vehicles for delivering TQ in oxidative-stress-related treatments [13]. Such approaches underscore the potential of thymoquinone-loaded nanoparticles as alternatives or adjuncts to conventional therapies, especially for conditions requiring long-term antioxidant intervention.

Nanotechnology in biomedicine offers new avenues to improve the bioavailability and efficacy of conventional therapies, including TQ. Techniques such as micelle nanoparticles, chitosan nanoparticles, and liposomes have been explored to enhance TQ’s oral bioavailability [18]. Nanochitosan (Cs NPs), a nanosized chitosan derivative, is particularly promising due to its biocompatibility and effectiveness as a drug delivery system [18]. Cs NPs also possess a higher surface area, which increases solubility [19]. Combining Cs NPs with TQ is hypothesized to enhance TQ’s bioavailability and stability, potentially amplifying its protective effects against age-related fertility issues in males. Notably, there is currently no research on the effects of the combined NCP treatment on testicular structure, sex hormones, and testicular anatomy in older individuals. Thus, using an aged rat model produced with D-galactose, the current study scrutinized the effects of TQ relative to NCPs on oxidant and antioxidant levels, testosterone levels, apoptosis, and testis structure in an aged rat model induced by D-galactose.

## 2. Materials and Methods

### 2.1. Chemicals

Sigma-Aldrich (St. Louis, MA, USA) provided the following: thymoquinone (TQ), dimethyl sulfoxide (DMSO), chitosan powder, and D-galactose (D-gal). The low molecular weight of Cs (90 to 95%, Oxford Lab Fine Chem LLP, Mumbai, India), sodium tripolyphosphate (TPP), NaOH, and TQ. All of these substances were of high purity.

### 2.2. Synthesis of Chitosan Nanoparticles and a Chitosan–Thymoquinone Nanocomposite (NCP)

Commercial chitosan (Cs) was dissolved in a 1% (*v*/*v*) aqueous acetic acid solution to prepare 1 g of the mixture, which was then stirred for two hours at ambient temperature. The pH of the Cs solution was adjusted to between 5.5 and 6 by adding NaOH solution drop by drop. Tripolyphosphate (TPP) (2.5 mg/mL) was then gradually added to the Cs solution at a ratio of 1:3 (*v*/*v*) while stirring for a full day, leading to the formation of solid Cs nanoparticles (Cs NPs). The solution was then centrifuged for 15 min at 6000 rpm. The separated Cs NPs were washed twice with a small amount of cold double-distilled water to remove unreacted substances, which was achieved by freeze-drying. Then, 0.1 g of Cs NPs were dispersed in 20 mL of double-distilled water, followed by stirring for 15 min at room temperature, and 0.1 g of TQ was added to the suspension. The mixture underwent ultrasonication for 30 min, followed by stirring overnight. The final product of Cs/TQ was filtered, washed, and dried at 40 °C (Supplementary Figure S2: the original ASCII (X, Y from the original data and the raw data source)).

### 2.3. Cell Culture and Cytotoxicity Assay

The H9C2 cell line, derived from rat myocardium, was supplied by Nawah Scientific Inc., located in Mokattam, Cairo, Egypt (Reference No. 300129). These cells were made available under a research-use license through CLS. Cultivation was conducted at 37 °C in a humidified incubator with 5% CO_2_. The growth medium used was Dulbecco’s Modified Eagle Medium (DMEM), which was enriched with 10% heat-inactivated fetal bovine serum, 100 units/mL penicillin, and 100 mg/mL streptomycin [20].

The Sulforhodamine B (SRB) assay was used to assess cell viability. A cell suspension of 5000 cells in 100 μL was seeded in 96-well plates with a complete medium and incubated for 24 h. Next, 100 μL of medium containing varying drug concentrations was added. After 72 h of drug exposure, the cells were fixed at 4 °C for one hour using 150 μL of 10% TCA. The TCA was removed, and the cells were rinsed five times with distilled water. Then, 70 μL of 0.4% (*w*/*v*) SRB solution was added, and cells were incubated in the dark at room temperature for 10 min. Following incubation, cells were washed three times with 1% acetic acid and left to air-dry overnight. Finally, the SRB stain was solubilized with 150 μL of 10 mM TRIS, and absorbance was measured at 540 nm using a BMG LABTECH^®^-FLUOstar Omega microplate reader [21].

### 2.4. Animals

Forty male Wistar rats (150 ± 5 g) were reared in well-ventilated plastic cages at room temperature. Both food and water were readily available to them. The food content was as follows: a yellow corn-soybean meal at 47%, limestone powder, dicalcium phosphate, crude soybean oil, wheat bran, sodium bicarbonate, table salt, and a mixture of vitamins and mineral salts. Every rat was kept on a 12:12 h light/dark schedule. After acclimating for 10 days, the rats were housed, and their weights were recorded weekly.

### 2.5. Experimental Design

After a ten-day acclimatization period, the rats were randomly allocated into four groups (n = 10 per group).

Control Group: These rats received a subcutaneous injection (0.5 mL) of physiological saline solution (0.9%) and an oral administration of physiological saline solution (0.9%) containing 0.1% DMSO daily for 56 days, starting from the first day and continuing until the end of the experiment.

D-gal Group: The rats received a subcutaneous injection of D-galactose at a dose of 120 mg/kg body weight, dissolved in 0.9% saline solution, following the protocol in [22]. Each rat was given a dosage of 0.5 mL daily for 42 days, starting on the 15th day and continuing until the end of the experiment.

D-gal + TQ Group: In addition to the same D-gal regimen as that of the D-gal group (120 mg/kg subcutaneously for 42 days from the 15th day onwards), these rats also received oral supplementation of thymoquinone (TQ) at a dose of 5 mg/kg B.W. [23] for the full 56 days of the experiment, starting from day one.

D-gal + NCP Group: Like the previous group, these rats were given 120 mg/kg of D-gal subcutaneously for 42 days, starting on the 15th day. Additionally, they received oral supplementation of nanochitosan–thymoquinone (NCP) at 5 mg/kg B.W. for the entire 56 days, beginning on the first day and continuing until the end of the study. 

### 2.6. Sampling

At the end of the experiment, the rats were anesthetized with diethyl ether, and blood samples were drawn from the orbital sinus. These samples were allowed to clot and then centrifuged at 5000 rpm for 10 min, with the resulting serum being stored at −20 °C for biochemical testing. Testicular tissue was rinsed in PBS (pH 7.4) and fixed in 4% paraformaldehyde for 48 h for histopathological and immunohistochemical assessments. Thin sections were subsequently fixed in 2.5% glutaraldehyde for ultrastructural examination. For biochemical analysis, the testes were washed with ice-cold 0.9% NaCl, homogenized in nine volumes of 0.1 M PBS (pH 7.4) to produce a 10% tissue homogenate, and centrifuged at 4000 rpm for 5 min at 4 °C. The supernatant was kept for biochemical assays, except for the assay of 3β-HSD activity. For the 3β-HSD assay, a separate portion was homogenized at 4 °C in a buffer containing 20% spectroscopic-grade glycerol, 5 mmol potassium phosphate, and 1 mmol EDTA; it was then centrifuged at 10,000× *g* for 30 min at 4 °C, with the supernatant being used for 3β-HSD activity measurement. Additional testicular samples were quickly frozen in liquid nitrogen and stored at −80 °C for gene expression analysis.

### 2.7. Oxidative Stress and Antioxidant Index Evaluation

The enzyme activity of SOD was assessed based on the method of Nishikimi et al. [24]. This method relies on the ability of SOD to inhibit the reduction of nitroblue tetrazolium (NBT) dye, a reaction facilitated by phenazine methosulfate. Catalase (CAT) enzyme activity was determined following Fossati et al. [25]. This assay is based on catalase’s ability to interact with a known amount of hydrogen peroxide (H_2_O_2_). After exactly one minute, a catalase inhibitor stops the reaction. The leftover H_2_O_2_ then reacts with 3,5-dichloro-2-hydroxybenzenesulfonic acid and 4-aminophenazone in the presence of horseradish peroxidase (HRP), producing a chromophore. The resulting color intensity is inversely related to the catalase concentration in the sample. The determination of malondialdehyde (MDA) followed the protocol of Ohkawa et al. [26] using a colorimetric assay kit (Biodiagnostic Company, Dokki, Giza, Egypt). This assay method relies on the reaction between thiobarbituric acid and malondialdehyde (MDA) under acidic conditions at 95 °C for 30 min, forming a thiobarbituric-acid-reactive product. The resulting pink color’s optical density is measured at 534 nm.

### 2.8. Testosterone Measurement

In serum and testicular tissue, testosterone levels were measured using an ELISA kit (ab108666, Abcam, Cambridge, UK). Briefly, 25 μL of standards, controls, or samples were added in duplicate to the ELISA plate, followed by 100 μL of the testosterone–HRP conjugate. After a 1 h incubation at 37 °C and three washes, 100 μL of TMB substrate was added and incubated in the dark for 15 min. After adding the stop solution, the absorbance was read at 450 nm, and the testosterone levels were calculated using a standard curve. There was an intra-assay CV of less than 10% and an inter-assay CV of less than 12%, demonstrating reliability. 

### 2.9. Steroidogenic Enzyme Assessment

The levels of 17β-HSD and LDH-C were determined using ELISA kits specifically for 17β-HSD (Catalog MBS2104946) and LDH-C (Catalog MBS2024970) (BioSource, Europe, Nivelles, Belgium. While 3β-HSD enzyme activity was estimated spectrophotometrically, one milliliter of testicular supernatant was combined with sodium pyrophosphate buffer, ethanol containing dehydroepiandrosterone, and bovine serum albumin, creating a 3 mL incubation mixture. To measure the 3β-HSD activity, 100 μL of NAD was added, and enzyme activity was recorded by recording the absorbance at 340 nm, using a blank without NAD as the control [27].

### 2.10. RT-PCR

Total RNA was isolated from testicular tissue using the Trizol reagent (Direct-zol™ RNA MiniPrep, catalog No. R2050, Zymo, Irvine, CA, USA) and quantified with a NanoDrop^®^ ND-1000 spectrophotometer(Thermo Scientific, Foster City, CA, USA). For cDNA synthesis, a 20 μL reaction was prepared using the SensiFast™ cDNA synthesis kit (Bioline, GmbH, Luckenwalde, Germany, catalog No. Bio-65053). Gene expression was then analyzed through real-time PCR using SYBR Green PCR Master Mix (2x SensiFast™ SYBR, Bioline, GmbH, Luckenwalde, Germany, catalog No. Bio-98002), with GAPDH serving as the reference gene (refer to Table S1 for details). The relative gene expression was calculated and normalized to GAPDH using the 2^−ΔΔCt^ method [28].

### 2.11. Histopathological Assessment

Testicular tissue samples were removed and quickly preserved for 2 days at room temperature in a 10% neutral buffered formalin solution. Standard techniques were used to process them for light microscopy. Tissue samples were placed for 30 min using increasing concentrations of ethanol, followed by three cycles of xylene to remove water; then, they were immersed in paraffin for two changes, forming a tissue block. The block was distended on glass slides after being cut to a thickness of 5 μm using a semi-automated rotating microtome (Leica RM 2125, Leica company, Wetzler, Germany). The slides were baked at 60 °C for one hour to remove extra paraffin. Ehrlich’s hematoxylin was used to stain these sections, which were then separated in acidified alcohol for 20 s, cleaned in distilled water, and rinsed in distilled water. Sections were transferred to tap water for 3 min (bluing), and afterward, the sections were stained in eosin for 1 min. After dehydrating in an increasing alcohol series, sections were cleaned in two xylene changes and mounted in DPX [29]. Johnsen’s score was applied to classify the stages of spermatogenesis [30].

### 2.12. Immunohistochemistry Analysis

Sections were stained using immunohistochemistry to detect p53-positive cells with anti-p53 antibodies. After deparaffinizing in xylene and hydrating through graded alcohols, sections were rinsed in PBS with 0.1% Tween 20. Antigens were retrieved by heating in sodium citrate solution (pH 6.0) at 90 °C for 20 min. Avidin/biotin blocking was applied, followed by incubation with monoclonal mouse anti-p53 antibodies (1:200 dilution) for 30 min at room temperature. After washing with PBS–Tween 20, peroxidase blocking was performed, and sections were treated with biotinylated antibodies and HRP-streptavidin for 30 min. The reaction was visualized using DAB, and sections were counterstained with Mayer’s hematoxylin, cleaned in xylene, and mounted in DPX [31].

### 2.13. Ultrastructure Assessment

Testis specimens were cut into thin sections (approximately 1 mm^3^) using a sharp knife for ultrastructure examination. They were then fixed in 2.5% glutaraldehyde, post-fixed in osmium tetroxide, buffered in 0.1M sodium cacodylate at 4 °C, dehydrated in ethyl alcohol grades, treated with propylene oxide, embedded in epoxy resin for an overnight period at 4 °C, and sectioned using an ultra-microtome [32]. Transmission electron microscopy was used to assess ultra-thin (50–100 nm thick) sections uploaded onto a copper grid and stained with lead citrate and uranyl acetate. Ultrathin sections were studied and observed at 160 kV using an electron microscope machine (JEOL JEM-2100, Tokyo. Japan) at Mansoura University in Egypt.

### 2.14. Statistical Analysis

We used GraphPad Prism 8 with one-way ANOVA and Tukey’s honestly significant difference (HSD) test for the statistical analysis. The mean ± standard error of the mean is how the data are presented, and significance is defined at *p* < 0.05.

## 3. Results

### 3.1. Identification and Characterization of the Designed Nanoparticles

**Figure 1:**
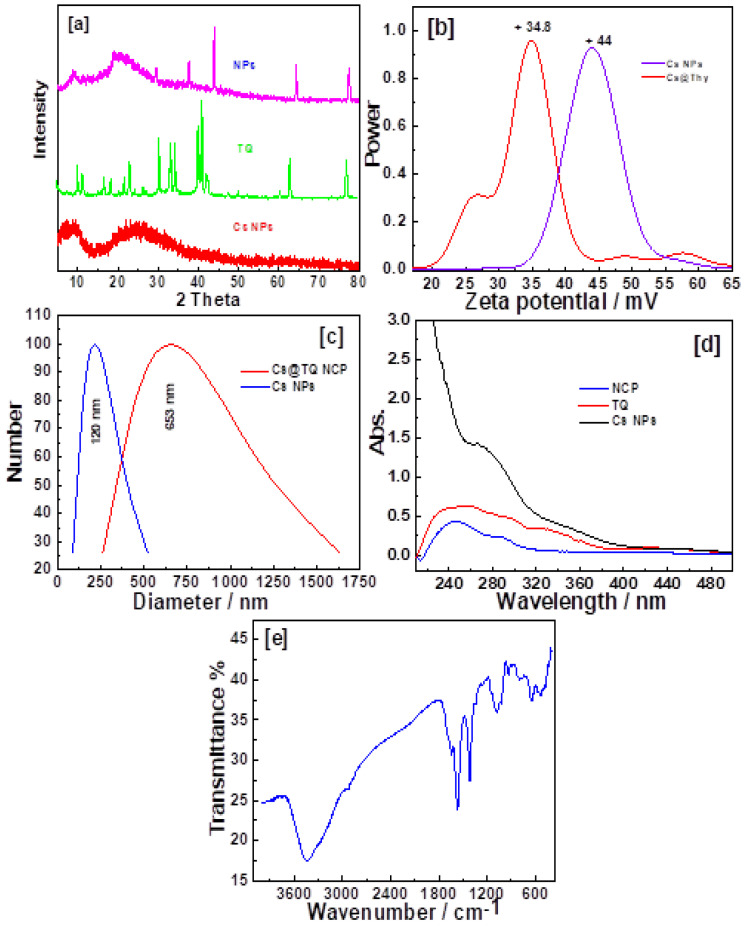
Characterization of the fabricated nanomaterials: (**a**) XRD patterns; (**b**) zeta potential; (**c**) dynamic light scattering; (**d**) UV−Vis spectroscopy; (**e**) FT−IR spectrum of Cs NPs.

X-ray diffraction (XRD) was measured using a Shimadzu 6000 (Kyoto, Japan). Figure 1a illustrates the formation of nano chitosan particles (Cs NPs). The XRD pattern appeared at 23° because of the idiosyncratic peak of the amorphous structure of the Cs NPs. The characteristic patterns of the synthesized TQ appeared at 9.2°, 12.4°, 14.95°, 16.1°, 21°, 22.8°, 25.8°, 26.9°, 30.4°, 33.9°, 36.65°, 37.66°, 43°, 43.8°, 45°, 50°, 52.2°, 62°, 64.3°, and 77.4°. The data indicated that the diffraction patterns at 33.9°, 43°, 50°, and 52.2° disappeared, while those at 36.65° and 45° showed reduced intensity. For the NCPs, new patterns emerged at 29.5°, 37.5°, 43.8°, 64°, and 77.6°. These findings confirmed the self-assembly formation between TQ and the surface of the Cs NPs. A diluted solution was used to investigate the stability and distribution of the samples, and a zeta potential experiment was performed in double-distilled water at room temperature. The electrostatic stability of the samples was indicated by the zeta potential values, with the Cs NPs showing +38.8 mV and +44 mV, though the value was typically around ±30 mV. The change in the zeta potential to +6 mV suggested dynamic interactions between TQ and the Cs NPs, which formed self-assembled Cs NPs. A dynamic light scattering (DLS) analysis showed particle sizes of 240 nm and 653 nm for Cs NPs (Figure 1c). The UV-Vis spectra of TQ and NCPs (Figure 1d) were recorded in double-distilled water at room temperature, showing characteristic peaks at 254 nm and 278 nm due to C=C (π-π*) transitions. Additionally, distinct peaks appeared at 295 nm, 329 nm, 360 nm, and 436 nm due to π-π* transitions in TQ, while the Cs NPs displayed peaks at 247 nm, 287 nm, 343 nm, and 425 nm. Cs NPs have distinguished bands owing to the amine group’s vibration at 3435 cm^−1^ and that of the hydroxyl groups at 3303 cm^−1^; CH_2_ group asymmetric stretching appeared at 2922 cm^−1^; while at 2852 cm^−1^, the unsymmetric stretching vibration appeared; the carbonyl group has bands at 1643 cm^−1^; NH_2_ and CN stretching and C-O have bands at 1563, 1400, and 1060 cm^−1^, respectively; and C-O-C appeared at 1155 cm^−1^.

#### SEM Micrograph

**Figure 2:**
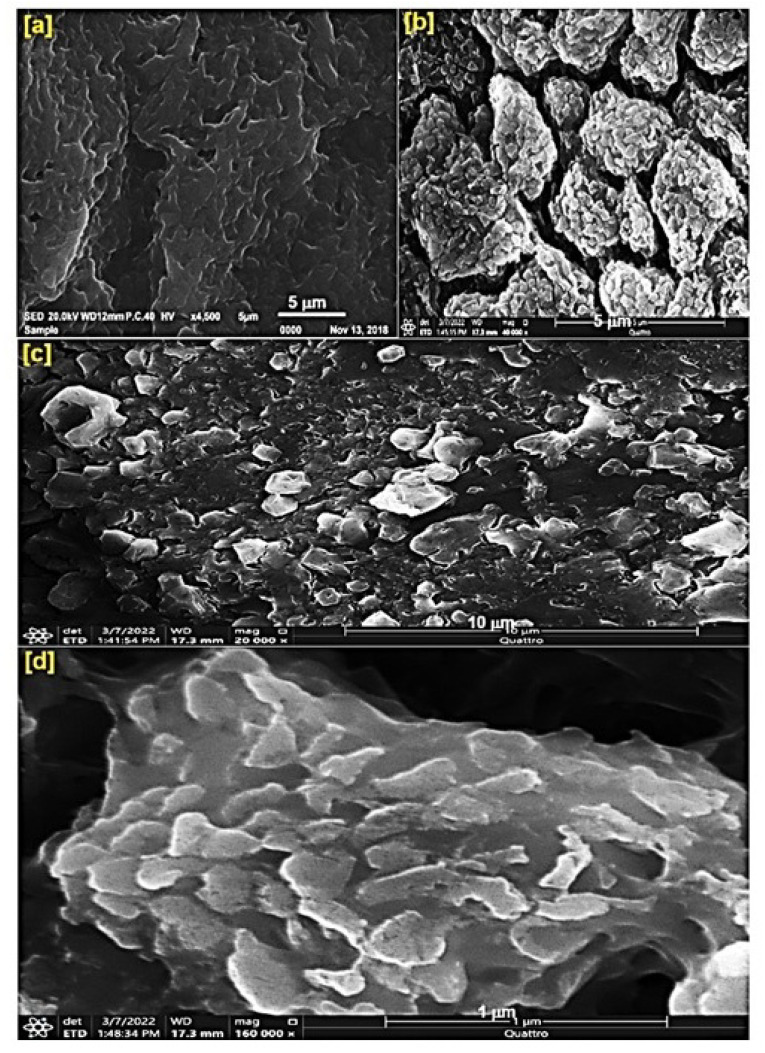

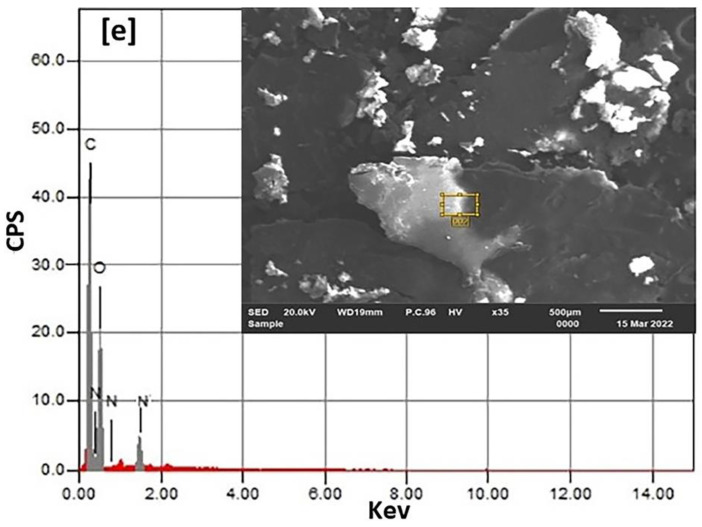
SEM micrograph of the fabricated nanomaterials: (**a**) Cs NPs (1 µm); (**b**–**d**) NCPs: 5 µm, 10 µm, and 1 µm; (**e**) EDX analysis.

Figure 2 presents the SEM micrographs of the Cs NPs, highlighting the surface morphology of the fabricated materials at various magnifications. An energy-dispersive X-ray (EDX) analysis was conducted to verify the formation of the fabricated materials. The micrographs reveal the surface characteristics of hybrid material. Cs NPs appeared as amorphous structures (Figure 2a), while the Cs NP images showed TQ loaded onto the surface of Cs NPs (Figure 2b,c). The overlapping of the two surfaces is evident, with TQ NPs being settled on the Cs NPs, forming the outer layer of the NCPs. EDX analysis confirmed the materials’ successful fabrication and the loading of TQ onto the surface of the Cs NPs (Figure 2d). The ratio of all elements (C, O, and N) was changed with NCPs in comparison with the Cs NPs. This information emphasizes the formation of NCPs (Table S2).

From the SEM micrograph and EDX analysis, the drug was set on the surface, and some amount was absorbed internally; also, the XRD patterns emphasized the loading process of TQ on the surface, and parts of it were incorporated internally, where the patterns of Cs NPs appeared. The NCP composite was formed as a self-assembly compound, facilitating the easy release of TQ with Cs NPs as a sole compound. The bio-properties of NCP, such as bioavailability and solubility, improved as a result of the Cs NPs as a bio surface. The nature of the binding between the Cs NPs and TQ, i.e., through physical binding via a self-assembly process, makes the release of TQ easier. The physical binding was discerned through the results of the EDX, XRD, and SEM micrograph, changing the zeta value of NCP compared to the Cs NPs alone.

### 3.2. Cytotoxicity Assay

**Figure 3:**
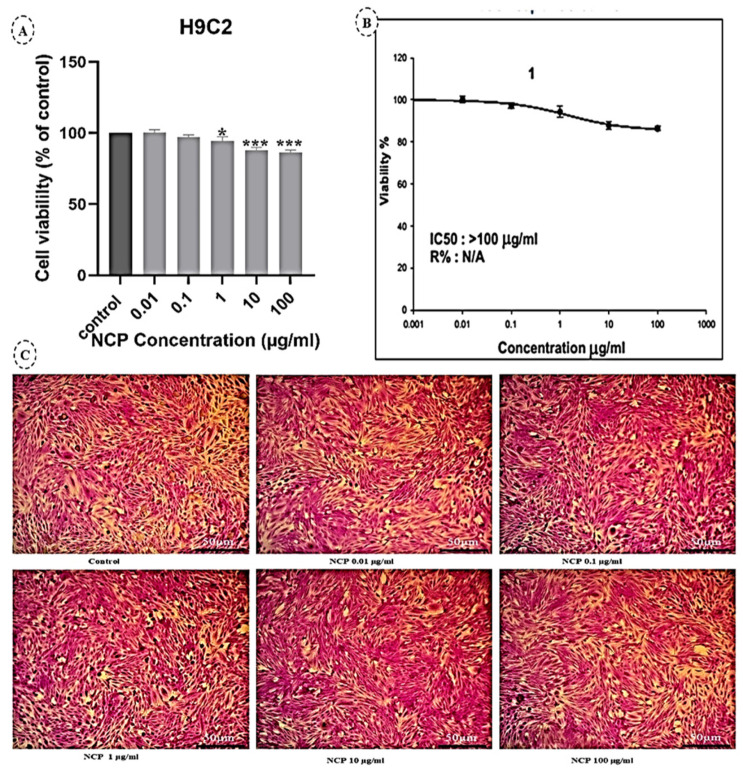
(**A**) Inhibition of cellular viability by NCPs (mean ± SE). *: *p* < 0.05; ***: *p* < 0.001. (**B**) Dose-response effect of NCPs on cellular viability (IC50). (**C**) H9C2 cells were incubated for 48 h in the presence or absence of NCPs (0.01, 0.1, 1, 10, and 100 µg/mL) on a cell invasion assay plate.

As shown in Figure 3A, exposure of rat heart/myocardium (H9C2) cells to increased concentrations of NCPs (0.01–100 µg/mL) for 24 h decreased cellular viability in a concentration-dependent manner. The IC50 value for the H9C2 cell line treated with NCPs was >100 µg/mL, as shown in Figure 3B. In addition, NCPs reduced the invasiveness of H9C2 cells at 100 µg/mL (non-toxic concentration), as represented in Figure 3C. NCPs significantly reduced the invasive potential of H9C2 cells. 

### 3.3. Body Weight

**Figure 4:**
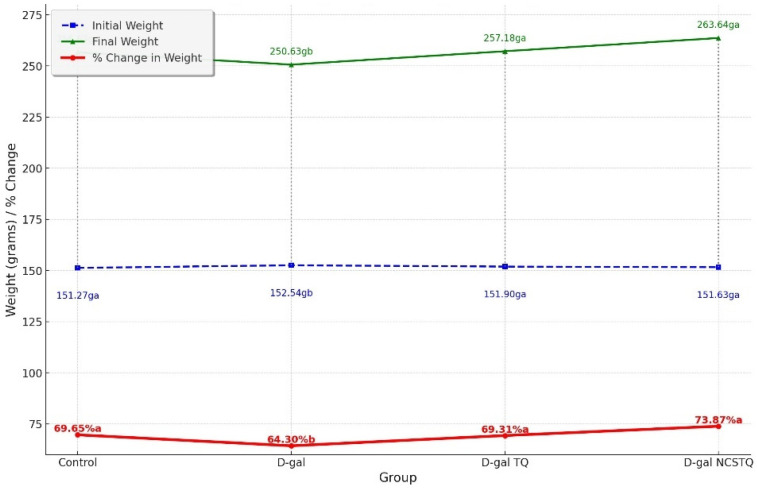
The effects of TQ and NCP administration on body weight and the percentage change in body weight. Data are shown as the mean ± SE. Different subscript letters indicate significant differences at *p* < 0.05.

The rats were weighed each week from the first week of the experiment until the end. There was an increase in body weight after administering TQ and NCPs compared with the initial body weight, as demonstrated in Figure 4. 

### 3.4. Testicular Antioxidant and Oxidative Stress Status

Concerning the serum levels of antioxidants (SOD, CAT) and the oxidative stress marker (MDA), our results demonstrate a substantial decline in serum SOD and CAT levels in the D-gal group in comparison with the control group. However, treatment with TQ or NCPs significantly increased the SOD and CAT levels relative to the D-gal group. The MDA levels, which indicate oxidative stress, were substantially higher in the aged rats in comparison with the controls. Treatment with TQ and NCPs significantly lowered the MDA levels, with NCPs having a more pronounced effect than TQ.

**Figure 5:**
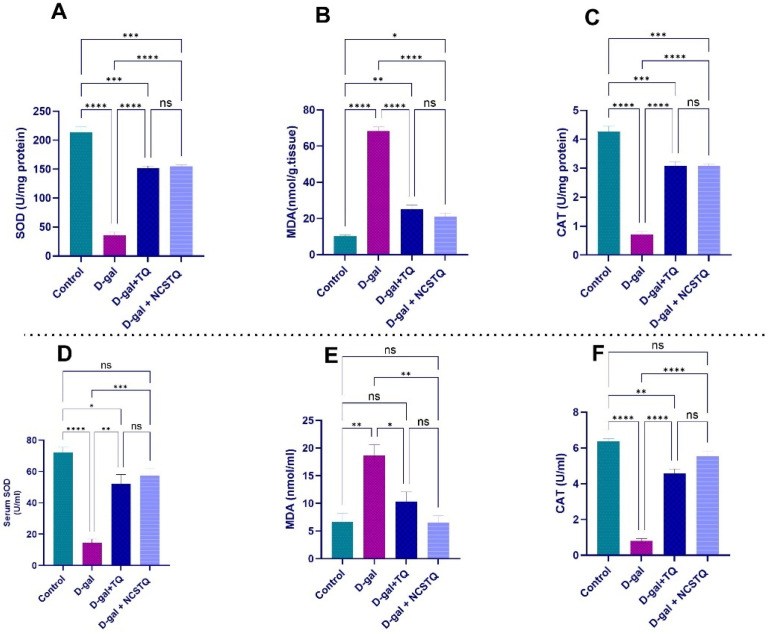
Effects of D-gal, TQ, and NCPs on SOD, MDA, and CAT levels in the serum and testicular tissues of rats. Panels (**A**–**C**) represent the levels of SOD, MDA, and CAT in the testicular tissues, respectively. Panels (**D**–**F**) display the corresponding serum levels of SOD, MDA, and CAT. Each bar represents the mean ± SE. * *p* < 0.05, ** *p* < 0.01, *** *p* < 0.001, **** *p* < 0.0001, and ns = not significant.

In testicular tissues, the aged rat group displayed a substantial decrease in SOD and CAT levels compared with the controls. Treatment with TQ or NCPs significantly elevated these antioxidant levels in the aged rat group. The MDA levels in testicular tissue were significantly higher in the D-gal group, indicating increased oxidative stress. While the TQ and NCP treatments reduced MDA levels, only NCP significantly inhibited lipid peroxidation. In comparison with the D-gal group, TQ did not significantly lower the MDA levels (Figure 5).

**Figure 6:**
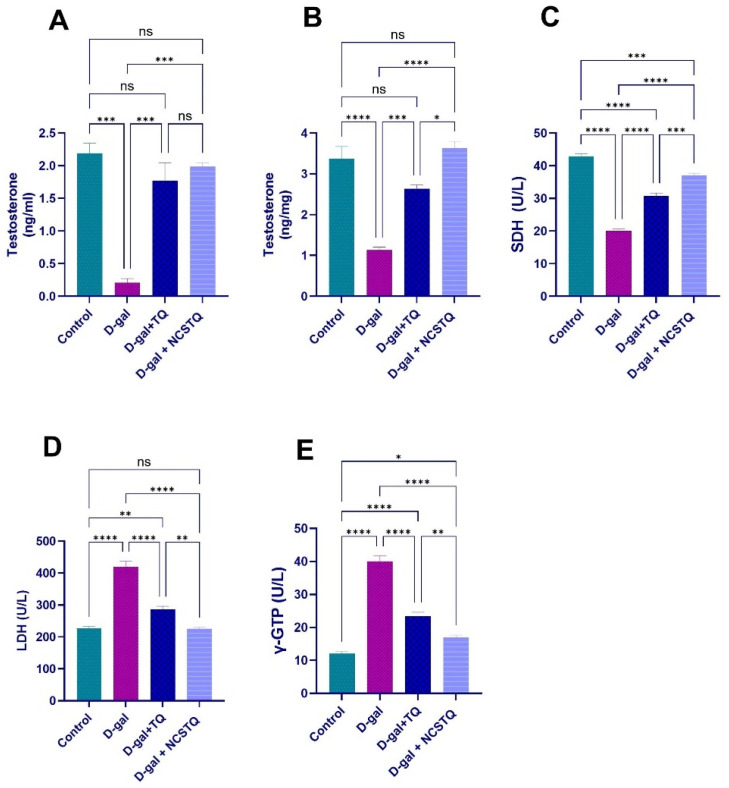
Effects of D-gal, TQ, and NCPs on testosterone levels and SDH, LDH, and γ-GTP activity in rats: (**A**) testicular testosterone; (**B**) serum testosterone; (**C**) succinate dehydrogenase (SDH); (**D**) lactate dehydrogenase (LDH); (**E**) gamma-glutamyl transpeptidase (γ-GTP). The data are expressed as the mean ± SEM. * *p* < 0.05, ** *p* < 0.01, *** *p* < 0.001, **** *p* < 0.0001, and ns = not significant.

The results illustrated in Figure 6 demonstrate that the D-gal treatment meaningfully decreased both testicular and serum testosterone levels while increasing markers of tissue damage and oxidative stress, such as LDH and γ-GTP activity, and decreasing SDH activity. Conversely, treatment with TQ or NCPs effectively restores testosterone levels. It normalizes enzyme activities, with NCPs showing a more pronounced protective effect in reducing oxidative stress and tissue damage than TQ. This suggests that NCPs may offer stronger protective benefits against the harmful effects induced by D-gal.

### 3.5. Steroidogenic Enzyme Activity

**Figure 7:**
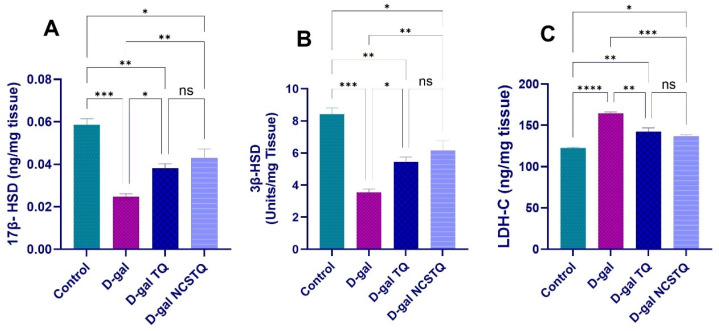
Effects of D-gal, TQ, and NCPs on testicular 17β-HSD, 3β-HSD, and LDH-C activity in rats: (**A**) testicular 17β-HSD; (**B**) testicular 3β-HSD; (**C**) testicular LDH-C. Data are expressed as the mean ± SEM. * *p* < 0.05, ** *p* < 0.01, *** *p* < 0.001, **** *p* < 0.0001, and ns = not significant.

Our findings indicate that the levels of 17β-HSD and 3β-HSD enzyme activity substantially decreased in the aged rat group in comparison with the control group. Conversely, the levels of 17β-HSD and 3β-HSD substantially improved in the TQ- and NCP-treated groups in comparison with the D-gal group. The activity level intensified in the D-gal group in comparison with the control group. However, the enzyme activity of LDH-C revealed a large decrease in the groups treated with D-gal/TQ and the D-gal/NCPs in comparison with the aged rat group. The group treated with D-gal/NCPs had the greatest decrease, as shown in Figure 7.

### 3.6. Testicular Histopathology 

**Figure 8:**
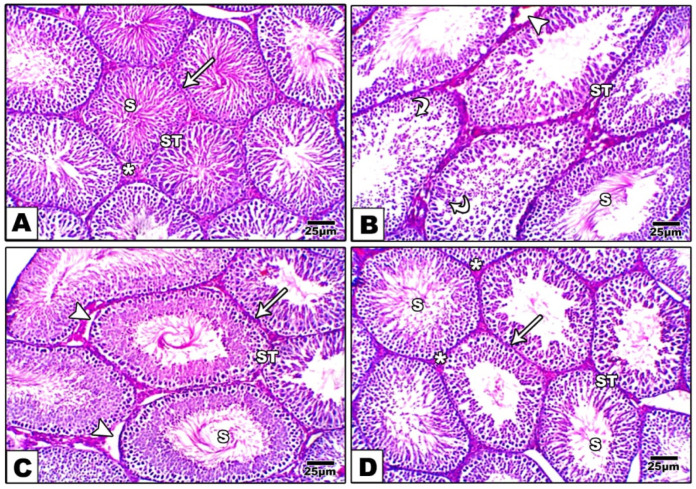
Photomicrographs of H&E staining of rat testis sections in different experimental groups and the control. (**A**) Normal control group showing normal testicular tissue histology with normal arrangement of spermatogonia and Sertoli cells and normal shape of seminiferous tubules (STs) resting on an intact basement membrane (arrow). Normal late-stage sperm (S) in the lumen and normal Leydig cell distribution (*). (**B**) The D-gal group showed degenerated STs, with numerous degenerated germ cells (curved arrows), absence of late-stage sperm (S), and separation (arrowhead) of basal and adluminal cellular compartments in some STs. (**C**) The D-gal TQ group shows a nearly normal ST structure with the separation (arrowhead) of basal and adluminal layers in some areas, as well as an intact basement membrane (arrow). (**D**) The D-gal NCP group shows slight amelioration of most seminiferous tubules (STs), with no noticed histopathological changes, normal late-stage sperm (S) in the lumen with a normal basement membrane (arrow), and Leydig cells (*). Magnification ×: 400.

Histopathological examination indicated that the normal control group showed normal testicular tissue histology with normal shapes of seminiferous tubules and normal arrangements of Sertoli cells and spermatogonia (Figure 8A). The D-gal group indicated degenerated ST, with numerous degenerated germ cells and the absence of late-stage sperm (Figure 8B). The treated groups showed improvements in testicular tissue with varying degrees (Figure 8C,D). The results indicate that the D-gal treatment significantly impairs testicular structure and spermatogenesis, as shown by the reduced Johnsen scores, decreased seminiferous tubule diameter and surface area, lower epithelial height, and increased thickness of the tunica albuginea. The control and D-gal + NCSTQ groups exhibit significantly better outcomes across these parameters, suggesting that NCSTQ effectively preserves or restores testicular health. The D-gal + TQ group also shows improvement compared with D-gal alone but to a lesser extent than NCSTQ. Overall, these findings highlight the protective effects of both TQ and NCSTQ treatments, with NCSTQ demonstrating the most consistent benefit in mitigating D-gal-induced testicular damage (Figure S2).

### 3.7. Immunohistochemistry of P53 

**Figure 9:**
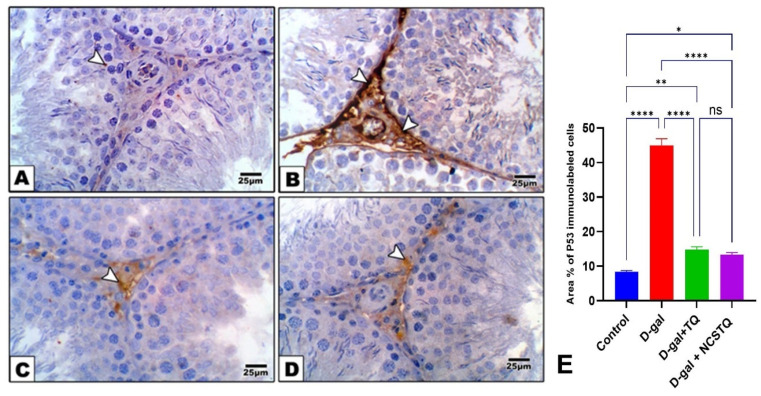
Immunohistochemical staining in testicular sections from the control and experimental groups using P53 immunostaining. (**A**) Normal control group: P53-immunolabeled cells were rarely present in the testes of control rats with a normal seminiferous tubule (ST) architecture. (**B**) D-gal group: This was identified by a slight brown staining increase, representing P53-immunolabeled cells as Leydig cells (thick arrows) and spermatogonial cells (thin arrow), suggesting an inflammatory response and a slight increase in apoptosis. (**C**) D-gal TQ group: P53-immunolabeled cells were moderately increased (arrow), indicating slight testicular section injury. (**D**) D-gal NCP group: A few cells showed positive expression for P53, indicating amelioration in testicular sections. The brown color indicates immunopositivity for P53 staining; ST: seminiferous tubule. LC: Leydig cells. Magnification: 40×. (**E**) Percentage area of P53-immunolabeled cells. Data are expressed as the mean ±SEM. * *p* < 0.05, ** *p* < 0.01, **** *p* < 0.0001, and ns = not significant.

Immunohistochemical staining of rat testis sections with P53 (tumor suppressor gene) exposed rare immunolabeling (weak staining) in the testes of the normal control rats with a normal seminiferous tubule (ST) architecture (A). In contrast, the D-gal group showed strong staining with P53, suggesting an inflammatory response and a slight increase in apoptosis (B). The D-gal TQ group showed that P53-immunolabeled cells were moderately increased, indicating slight testicular injury (C). The D-gal NCP group showed that a few cells were positive for the expression of P53, indicating amelioration in testicular sections (D), as shown in Figure 9.

### 3.8. Transmission Electron Microscopic Investigation

**Figure 10:**
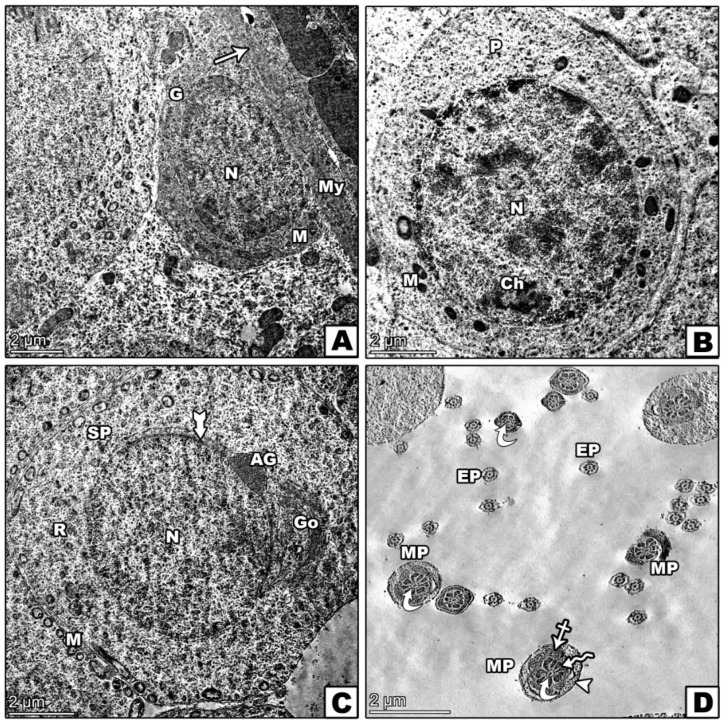
Transmission electron microscopy (TEM) of the effects of D-gal, TQ, and NCPs on the seminiferous tubules from the testes of the control group showing (**A**) normal spermatogonia (G) with a large spherical nucleus (N) and mitochondria (m) resting on a regular basement membrane (arrow) alongside a myoid cell with a flattened nucleus (My). (**B**) A primary spermatocyte (P) with a rounded nucleus (N) containing heterochromatin clumps (Ch) and normal mitochondria (M). (**C**) A round spermatid (SP) with an euchromatic nucleus (N), a well-formed acrosomal cap (tailed arrow), an acrosomal granule (AG), and a flattened Golgi body (Go). Mitochondria (M) are peripherally arranged with free ribosomes (R). (**D**) Transverse sections of the sperm tail show the middle piece (MP) with central microtubules (curved arrows), outer dense fibers (zigzag arrow), a mitochondrial sheath (crossed arrow), and a plasma membrane (arrowhead). In contrast, the end piece (EP) includes a central axoneme within a plasma membrane.

**Figure 11:**
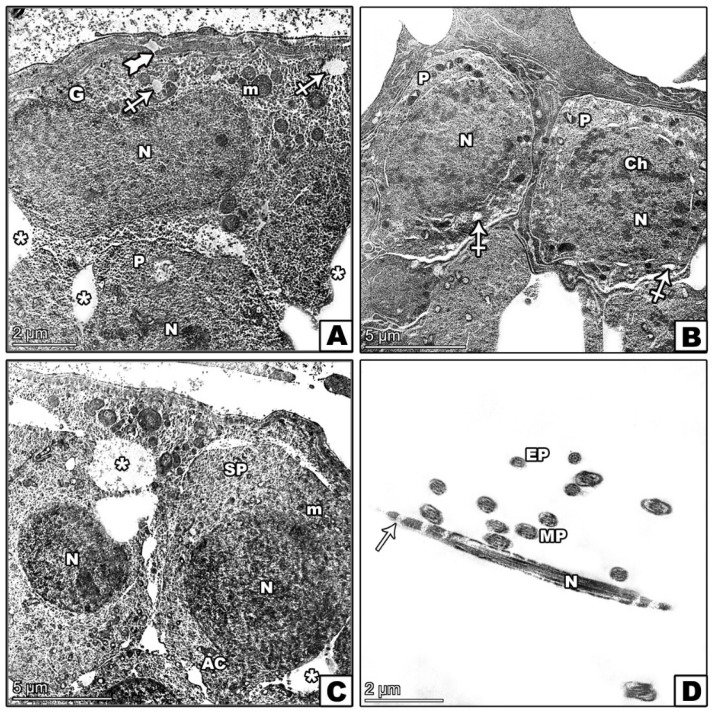
Transmission electron microscopy (TEM) of the effects of D-gal, TQ, and NCPs on the seminiferous tubules from the testes from the D-gal group showing (**A**) spermatogonia (G) with an irregular membrane partially detached from the basement membrane (arrowhead), an oval nucleus (N), and enlarged mitochondria (m) with vacuoles (crossed arrow). A primary spermatocyte (P) with an irregular nucleus (N) and increased cell-to-cell junctions (*). (**B**) Two primary spermatocytes (P) with distorted shapes, large nuclei (N) with peripheral heterochromatin, and cytoplasm containing mitochondria (m) and vacuoles (crossed arrow). (**C**) A round spermatid (SP) with an eccentric nucleus (N), abnormally positioned mitochondria (m), widened intercellular spaces and necrotic debris in the intervening spaces (*), and a degenerated acrosomal cap (AC). (**D**) Shrinkage of the middle (MP) and end pieces (EP) of sperm, with cross-sections showing a well-developed sperm head with a nucleus (N) and vacuolated acrosomal cap (arrow). Vacuoles are present in the cytoplasm.

**Figure 12:**
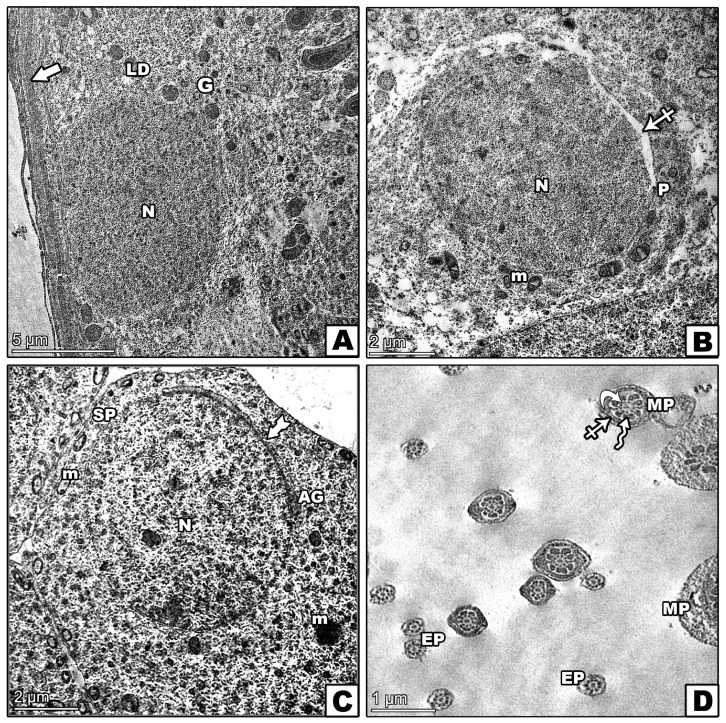
Transmission electron microscopy (TEM) of the effects of D-gal, TQ, and NCPs on seminiferous tubules from the testes of the D-gal TQ group showing (**A**) spermatogonia (G) with a large, irregular nucleus (N) and cytoplasm containing normally shaped mitochondria (m) but with an abnormal distribution, resting on a thin basement membrane (thick arrow) with numerous lipid droplets (LDs). (**B**) A primary spermatocyte (P) with an irregularly shaped nucleus (N), small mitochondria (m), and visible vacuoles (crossed arrow). (**C**) A round spermatid (SP) with a euchromatic nucleus (N), some mitochondria with abnormal shapes scattered throughout the cytoplasm (m), an acrosomal cap (tailed arrow), and an acrosomal granule (AG). (**D**) Middle (MP) and end pieces (EP) of sperm, each containing a central axoneme (curved arrow), varying sizes of the middle piece with a moderately normal axoneme enclosed by nine thick, dense fibers (zigzag arrow), and mitochondrial sheaths (crossed arrow).

**Figure 13:**
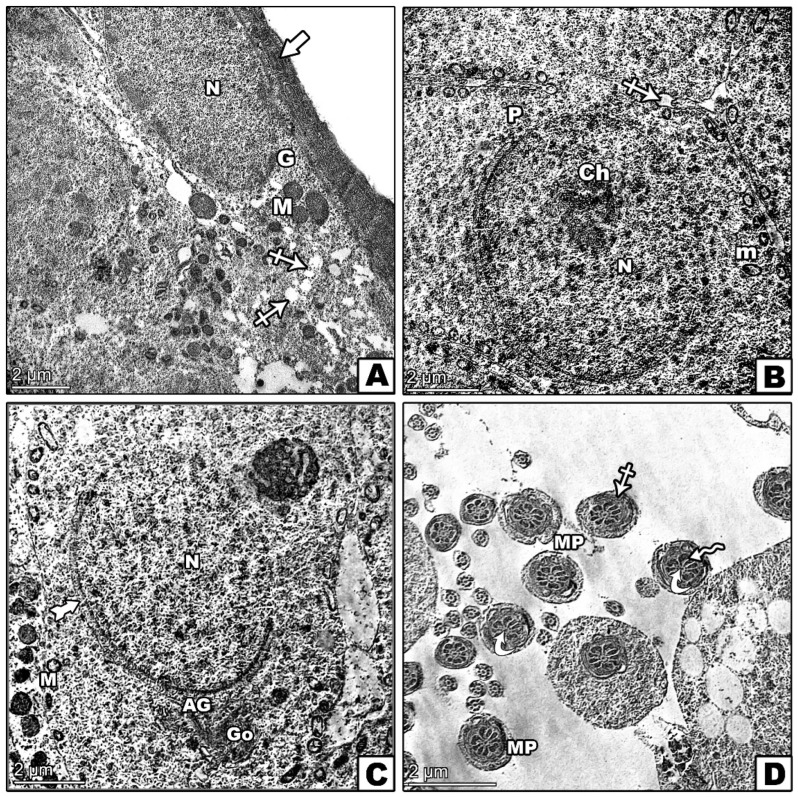
Transmission electron microscopy (TEM) of the effects of D-gal, TQ, and NCPs on (**A**) spermatogonia (G) with a normal nucleus (N). An electron micrograph of several sections of seminiferous tubules taken from the testes of the D-gal NCP group; cytoplasm containing mitochondria (M) with different sizes, cells resting on a regular basement membrane (thick arrow), and multi-vacuoles (V) still appear. (**B**) The primary spermatocyte (P) has rounded nuclei (N) containing clumps of heterochromatin (Ch) that are scattered all over the nucleoplasm, with the appearance of disorganized mitochondria (m) and few vacuoles (crossed arrow). (**C**) The spermatid (SP) has a euchromatic nucleus (N) and a well-formed acrosomal cap (tailed arrow). Mitochondria (m) appear near one pole of the nucleus, forming a flattened Golgi body (Go). (**D**) Most middle pieces (MPs) of sperms in the lumen have a normal central axoneme (curved arrows) with the presence of swallowed middle pieces with mitochondrial sheaths (crossed arrow) and dense fibers (zigzag arrow).

The control group exhibited a normal seminiferous tubule structure, healthy spermatogonia on an intact basement membrane, and primary spermatocytes with rounded nuclei (Figure 10A,B). The lumen contained well-formed spermatids (Figure 10C,D). In contrast, the D-gal group illustrated significant abnormalities, including irregular spermatogonia, which were partially detached from the basement membrane, and distorted primary spermatocytes (Figure 11A,B). Abnormal spermatids with eccentric nuclei and mispositioned mitochondria were also observed (Figure 11C,D). The D-gal TQ group displayed moderate improvements, with thinner basement membranes and lipid droplets present, but there were irregular nuclei in primary spermatocytes, and varying middle pieces of sperm persisted (Figure 12A–D). The D-gal NCP group showed a nearly normal tubule structure, with regular basement membranes, rounded nuclei in primary spermatocytes, and mostly normal spermatids (Figure 13A–D).

### 3.9. Regulators of Longevity, Stress Response, Mitochondrial Function, Antioxidant Defense, and Reproductive Health Gene Expression

**Figure 14:**
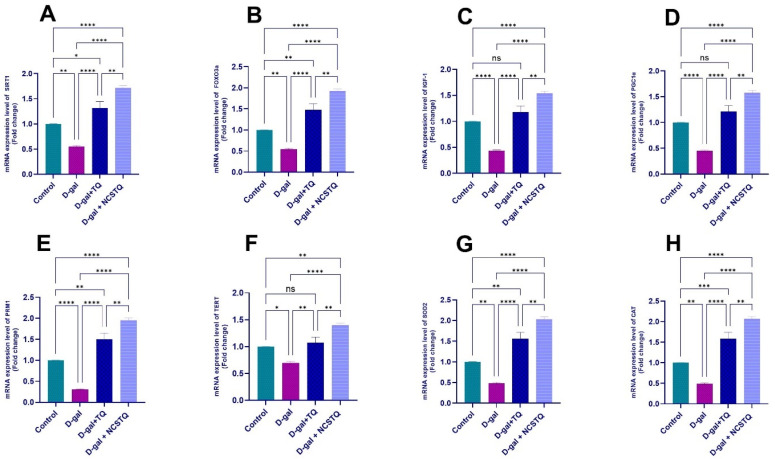
Effects of D-gal, TQ, and NCPs on the mRNA expression levels of (**A**) *SIRT1*, (**B**) *FOXO3a*, (**C**) *IGF-1*, (**D**) *PGC1α*, (**E**) *PRM1*, (**F**) *TERT*, (**G**) SOD2, and (**H**) CAT in rat testicular tissue. The data represent the mean ± SEM. * *p* < 0.05, ** *p* < 0.01, *** *p* < 0.001, **** *p* < 0.0001, and ns = not significant.

Figure 14 illustrates the mRNA expression levels of key genes implicated in aging and the stress response—*SIRT1*, *FOXO3*, *IGF-1*, *PGC1α*, *PRM1*, *TERT*, SOD2, and CAT—across different experimental groups. The control group shows baseline expression levels for all genes. The D-gal group, which was subjected to aging induction, exhibited significantly reduced expression of all genes in comparison with the control, indicating the negative impact of D-galactose on these critical protective genes. Treatment with thymoquinone (D-gal + TQ group) partially restored the expression levels of these genes, though not to the control levels, suggesting a protective but incomplete impact of thymoquinone on D-gal-induced aging. Notably, the group treated with thymoquinone-loaded chitosan nanoparticles (D-gal + NCP group) presented a noteworthy upsurge in the expression of all genes associated with both the D-gal and D-gal + TQ groups, with levels approaching or exceeding those of the control group. This indicates that NCPs offer a more robust protective effect against aging at the molecular level, particularly in maintaining or enhancing gene expression for stress resistance and mitochondrial function.

### 3.10. Multivariate Assessments

The heatmap displays the Pearson correlation coefficients among eight variables: SOD, MDA, CAT, testosterone, LDH, 3β-HSD, 17β-HSD, and γ-GTP. Red indicates strong positive correlations, while blue shows strong negative correlations. Most variables are strongly positively correlated, with coefficients close to 1.00. For example, LDH correlates highly with testosterone, 3β-HSD, 17β-HSD, and γ-GTP. In contrast, SOD shows strong negative correlations with several variables, including LDH and γ-GTP. This suggests that there are complex interrelationships among these biochemical markers (Figure S3). The PLS-DA score plot shows the distribution of observations across two components (PLS-DA Component 1 and Component 2) for classes labeled Class 0 and Class 1. The plot illustrates a partial separation between the two classes, indicating that the PLS-DA model can differentiate between them to some extent (Figure S4).

The volcano plot indicates that several genes in the testes are significantly differentially expressed. Genes with a log2 fold change outside the threshold values (indicated by the vertical green dashed lines) and a *p*-value below the significance threshold (represented by the horizontal blue dashed line) are marked as significant. These significant genes, shown as red points, include up- and downregulated genes. The presence of these genes suggests notable alterations in gene expression that could be crucial for understanding the underlying biological processes in the testes (Figure S5). The figure indicates a plot of VIP scores and a heatmap of key biomarkers. The VIP scores indicate that biomarkers such as abnormal sperm morphology, testosterone, SOD, and LH are most important in the model, with scores above 1. The heatmap reveals varying expression levels of biomarkers across different treatment groups, including the control group, the group with formaldehyde exposure, and groups with various dosages of treatments. Notably, formaldehyde exposure alters biomarker expression, with some treatments potentially reversing these effects, highlighting their significance in reproductive health and toxicity response (Figure S6).

## 4. Discussion

In this investigation, we hypothesized the protective role of TQ in comparison with NCPs against aging induced by D-gal in male rats. The aged rat group had a substantial decline in body weight in comparison with the control group. Several researchers argued that D-gal injection diminished the body weight in aged rats compared with control rats [33]. On the contrary, the body weight of the elderly rats was drastically higher in the TQ group than in the D-gal group after administration. This finding is in agreement with the results of [34], in which it was demonstrated that TQ increased the body weight of rats in comparison with that of rats with diabetes.

Several body organs’ oxidative stress changes are linked to aging, including the testes [35]. This study indicated that D-gal administration led to a substantial reduction in the CAT and SOD activity. However, treatment with TQ and NCPs significantly elevated the CAT and SOD levels in comparison with the aged rat group. Conversely, the MDA levels in serum and testicular tissue were substantially greater in the aged group than in the control group. TQ or NCPs notably reduced the MDA levels when assessed in the aged group, with NCPs showing a more pronounced effect than TQ, particularly in lowering the serum and testicular MDA levels. Several studies have highlighted the defensive consequences of TQ alongside the oxidative harm induced by D-gal and other stressors. In contrast to the aged rat group, TQ noticeably raised the total antioxidant capacity (TAC) and decreased the testicular MDA levels [36].

Similarly, mice exposed to cadmium toxicity had their testicular SOD activity boosted, and their MDA levels decreased when given TQ [37]. Rats suffering from ischemia-reperfusion injury showed a marked improvement in SOD activity and a reduction in MDA levels after receiving TQ [38]. TQ condensed the levels of MDA and boosted the activity of SOD and catalase, protecting the testes of hypothyroid rats [39]. Concerning doxorubicin-induced toxicity, TQ decreased the MDA levels and intensified testicular antioxidant enzyme levels. We are unaware of any research examining how NCPs affect antioxidant enzymes and testicular oxidative stress in older adults [40].

Exposure of rat myocardium (H9C2) cells to increasing concentrations of NCPs (0.01–100 µg/mL) for 24 h reduced cell viability in a concentration-dependent manner, with an IC50 value exceeding 100 µg/mL. NCPs also significantly inhibited the invasive potential of H9C2 cells. Comparing our result with other studies that used the free drug, it was found that thymoquinone (TQ) has only been studied for its protective and cytotoxic effects across various cell types. In a study on doxorubicin-induced cardiotoxicity, TQ pre-treatment in H9c2 cardiomyocytes reduced cell death and apoptosis caused by doxorubicin (Dox), highlighting its cardio-protective potential. Non-toxic concentrations of TQ (<10 μM) were identified using the CCK-8 assay. TQ significantly reversed Dox-induced reductions in cell viability and death in a dose-dependent manner [41]. TQ also demonstrated anti-cancer properties. TQ (1–100 μM) inhibited the viability of lung, liver, colon, melanoma, and breast cancer cells, with HepG2 liver cells being the most sensitive (IC50 = 34 μM) [42]. It was also reported that the TQ’s anti-proliferative effect on ovarian cancer cells (IC50 = 6.0 μg/mL) without harming normal hepatocytes [43]. Similarly, the TQ’s cytotoxicity against HeLa cervical carcinoma cells, with IC50 values decreasing over time (5.93 mg/mL at 24 h to 2.80 mg/mL at 72 h). Both trypan blue and MTT assays confirmed TQ’s dose- and time-dependent cytotoxicity, with morphological changes indicating cell death at higher concentrations (10–30 mg/mL) [44]. In addition, in glioma cells, it was the decreased cell viability with increasing TQ concentrations (2.5–200 μM), with an IC50 of 72 μM. These findings collectively emphasize TQ’s potential as a cardio-protective and anti-cancer agent [45]. In addition, Chitosan, a biocompatible and biodegradable polymer, positively impacts cell cultures such as H9C2 cardiomyoblasts and MG-63 osteoblast-like cells. It enhances adhesion and proliferation in H9C2 cells, aiding myocardial tissue engineering [46], and supports differentiation and mineralization in MG-63 cells for bone tissue engineering [47]. Chitosan’s antimicrobial properties prevent contamination, and its hydrogel and nanoparticle forms facilitate drug delivery. However, high concentrations may cause cytotoxicity, requiring careful optimization.

This study found that 17β-HSD and 3β-HSD activities were significantly decreased in the aged group in comparison with the controls, which was consistent with the findings from [48], who also reported decreased steroidogenic enzyme activity in aged rats. Conversely, TQ significantly increased these enzymes’ activity, supporting the results of [49], who observed similar increases in black-seed-oil-treated rats. Additionally, LDH-C activity was significantly elevated in the aged rats, aligning with the findings of [50]. The TQ treatment substantially reduced LDH activity, which was consistent with the findings of [51]. The NCP treatment also significantly decreased the LDH-C levels in comparison with the aged group, which was similar to the results of Wardani et al. [52]. However, no one has ever looked at the impacts of NCPs on 17β-HSD, 3β-HSD, and LDH enzyme activity in the testes of aged male rats. This work highlights the detrimental impact of D-galactose (D-gal) on testosterone levels and oxidative stress markers, showing significant reductions in testosterone and SDH activity alongside increases in LDH and γ-GTP activity, which indicates oxidative stress and tissue damage [53]. The administration of thymoquinone (TQ) and nano chitosan–thymoquinone (NCP) effectively counteracted these effects, with both treatments restoring testosterone levels and normalizing enzyme activities. Notably, NCPs demonstrated a stronger protective effect than TQ alone, which was likely due to the enhanced bioavailability and stability provided by the nano-chitosan carrier [54]. These data indicate that NCPs could be a more successful treatment strategy for mitigating oxidative injury and age-related dysfunctions, which is consistent with the existing literature on the benefits of nanotechnology in enhancing antioxidant efficacy [55]. The present study demonstrated significant degeneration in the seminiferous tubules of the D-gal group, including numerous degenerated germ cells, the absence of late-stage sperms, and disorganized cellular compartments, aligning with previous findings on aging-related testicular degeneration [3]. The TQ treatment improved the structure of seminiferous tubules, showing fewer histopathological changes, which was consistent with earlier studies that reported TQ’s protective effects on testicular tissue [36]. The NCP group exhibited even greater histological improvement, suggesting a stronger protective effect against D-gal-induced damage.

Further analysis revealed a substantial upsurge in apoptotic protein P53 expression in aged rats, indicating increased apoptosis. The TQ and NCP treatments reduced P53 expression, aligning with their roles in protecting against cellular damage [56]. Additionally, a microscopic examination showed that D-gal caused a maturation arrest in spermatogenesis, with germ cells only maturing to the spermatogonia stage, which was improved by TQ and further enhanced by the NCP treatment. This study is among the first to observe the protective impact of NCPs on testicular ultrastructure in aged rats, revealing its potential for mitigating age-related testicular damage more effectively than TQ alone.

Thymoquinone (TQ), a bioactive compound from *Nigella sativa*, is known for its antioxidant, anti-inflammatory, and anti-apoptotic assets. It reduces oxidative stress by enhancing SOD2 and CAT activity and scavenging free radicals [57]. In our study, the TQ treatment partially restored the expression of several key genes downregulated by D-galactose, including SIRT1, FOXO3a, IGF-1, PGC1α, TERT, SOD2, and CAT. These results align with those of previous studies demonstrating TQ’s ability to protect against oxidative injury and improve mitochondrial utility [58]. SIRT1 plays a critical role in promoting longevity and regulating cellular stress responses. It is known for deacetylating proteins that contribute to cellular regulation, including p53, NF-κB, and FOXO transcription factors, which are crucial for the interplay between oxidative stress and cellular aging [59]. In this study, SIRT1 expression was drastically reduced in the aged rat group, reflecting the adverse effects of D-galactose on cellular health. However, its expression was partially restored in the D-gal + TQ treated rats and substantially upregulated in the D-gal + NCP treated rats, demonstrating NCPs’ strong protection against D-gal-induced oxidative injury and aging. FOXO3a regulates oxidative stress resistance, apoptosis, and DNA repair genes, making it another important longevity gene [60]. Expression of FOXO3a was markedly reduced in the aged rats, which was consistent with its role in mitigating oxidative damage and promoting cellular survival under stress. The substantial improvement in FOXO3a expression in the NCP group underscores the enhanced protective capacity of NCPs, which is likely through the activation of FOXO3a-mediated pathways that enhance cellular resilience. IGF-1 is crucial for growth, development, maintaining muscle mass, and regenerative processes [61]. The observed decrease in IGF-1 expression in the aged rat group is indicative of impaired growth signaling pathways, which are often seen in aging. The recovery of IGF-1 expression in the D-gal + TQ group and its further enhancement in the D-gal + NCP group suggest that these treatments help maintain anabolic processes that are essential for tissue repair and longevity. PGC1α is pivotal in mitochondrial biogeny and energy metabolism, particularly in tissues with high energy demands, such as the brain and muscle [62]. The reduction in PGC1α expression in the D-gal group reflects mitochondrial dysfunction, a hallmark of aging. The enhanced expression of PGC1α in the NCP group suggests that this treatment helps preserve mitochondrial function and energy homeostasis, which is critical for delaying the aging process and maintaining cellular vitality. TERT, the catalytic subunit of telomerase, is accountable for maintaining telomere length, thereby preserving genomic stability and cellular lifespan [63]. The significant decrease in TERT expression in aged rats aligns with accelerated telomere shortening and cellular aging. The restoration and enhancement of TERT expression in the NCP group highlight the potential of this treatment to protect against telomere attrition, thereby promoting cellular longevity. The antioxidant enzymes SOD2 and CAT play vital roles in mitigating oxidative stress by detoxifying ROSs. After exchanging superoxide radicals for hydrogen peroxide, SOD2 and CAT break it down into oxygen and water to prevent oxidative damage [64,65]. The decreased expression of these enzymes in the D-gal group indicates impaired antioxidant defenses. The significant upregulation of both SOD2 and CAT in the NCP group suggests that NCP treatment effectively enhances the cellular antioxidant capacity, reducing oxidative stress and its associated damage. PRM1 is essential for properly packaging DNA in sperm, a critical process for maintaining sperm integrity and male fertility [66]. The decreased expression of PRM1 in the D-gal group reflects impaired sperm chromatin structure, leading to reduced fertility. The significant restoration of PRM1 expression in the NCP group indicates that this treatment supports reproductive health by preserving sperm DNA integrity, potentially counteracting the negative reproductive effects of aging. However, meaningful protective effects were demonstrated in the group treated with thymoquinone-loaded chitosan nanoparticles (NCPs). Chitosan nanoparticles enhance the bioavailability and stability of TQ, allowing for more efficient cellular uptake and sustained release [67,68]. This enhanced delivery system likely contributed to the more substantial upregulation of protective genes in the NCP group in comparison with TQ alone. For instance, the SIRT1 and FOXO3a expression levels in the NCP group were higher than in the TQ group, suggesting that NCPs offer superior protection against aging by activating pathways involved in stress resistance and longevity. The observed increase in IGF-1 expression in the NCP group also suggests that this treatment supports anabolic processes and tissue maintenance more effectively than TQ alone. Previous studies have highlighted the function of IGF-1 in counteracting muscle atrophy and promoting regenerative processes, which are crucial for maintaining physiological function during aging [69]. The enhanced expression of PGC1α in the NCP group further indicates improved mitochondrial biogenesis and function, which are critical for energy metabolism and delaying age-related decline [70].

Importantly, TERT expression was significantly higher in the NCP group, suggesting that NCPs may offer protective effects against telomere shortening, a hallmark of cellular aging [71]. The upregulation of SOD2 and CAT in the NCP group reflects a robust enhancement of the antioxidant defense system, which is likely due to TQ’s improved delivery and sustained action within the cellular environment [72,73]. Lastly, PRM1, a gene essential for proper sperm DNA packaging and male fertility, showed significant upregulation in the NCP group, indicating that NCPs might be particularly effective in preserving reproductive health during aging. The superior protection offered by NCPs compared with TQ alone is consistent with studies showing enhanced therapeutic efficacy of bioactive compounds when delivered via nanoparticle systems [74]. When comparing these findings with those of other studies, the data are in agreement with research demonstrating the defensive impact of TQ against oxidative injury and aging [59,60]. However, the enhanced outcomes observed with NCPs in this study highlight the ability of nanoparticle-based delivery systems to significantly enhance the bioavailability and efficiency of natural compounds such as TQ. This suggests that NCPs could be a promising therapeutic approach for combatting aging and related diseases more effectively than TQ alone.

## 5. Conclusions

This study demonstrates that thymoquinone (TQ), particularly when delivered via chitosan nanoparticles (NCPs), offers a significant defensive impact against D-galactose-induced aging in rat testes. NCPs demonstrated superior efficacy over TQ alone in reducing oxidative stress, preserving the testicular structure, restoring testosterone levels, and improving mitochondrial function and stress resistance gene expression. Considering these findings, NCPs could be an attractive treatment strategy for mitigating age-related reproductive dysfunction and oxidative damage. Future research should further elucidate how NCPs’ protective impacts can be applied and should explore potential applications in clinical settings.

## Data Availability

Data are available upon request.

## Supplementary Materials

The following supporting information can be downloaded at: https://www.mdpi.com/article/10.3390/pharmaceutics17020210/s1, Figure S1: Quantitative analysis of H9C2 cell invasiveness at different NCP concentrations; Figure S2: Comprehensive Heatmap of Pearson Correlation Coefficients Among Various Biomarkers.; Figure S3: The PLS-DA (Partial Least Squares Discriminant Analysis) score plot presented the separation between two different classes (Control and D-gal groups) based on the first two components derived from the PLS-DA model; Figure S4: Volcano Plot of differentially expressed genes in testis; Figure S5: VIP Scores and Heatmap of Key Biomarkers. Table S1: Primer Sequences. Table S2: EDX (Elements Composition Data). Table S3: Quantitative Analysis of H9C2 Cell Invasiveness at Different NCP Concentrations.
