# Correction: Kasem et al. Thymoquinone-Loaded Chitosan Nanoparticles Combat Testicular Aging and Oxidative Stress Through SIRT1/FOXO3a Activation: An In Vivo and In Vitro Study. *Pharmaceutics* 2025, *17*, 210

**DOI:** 10.3390/pharmaceutics18050597

**Published:** 2026-05-14

**Authors:** Enas A. Kasem, Gehan Hamza, Nagi M. El-Shafai, Nora F. Ghanem, Shawky Mahmoud, Samy M. Sayed, Mohammed Ali Alshehri, Laila A. Al-Shuraym, Heba I. Ghamry, Magdy E. Mahfouz, Mustafa Shukry

**Affiliations:** 1 Faculty of Science, Zoology Department, Kafrelsheikh University, Kafrelsheikh 33516, Egyptmagdymahfouz200188@gmail.com (M.E.M.); 2 Nanotechnology Center, Chemistry Department, Faculty of Science, Kafrelsheikh University, Kafrelsheikh 33516, Egypt; 3 Department of Physiology, Faculty of Veterinary Medicine, Kafrelsheikh University, Kafrelsheikh 33516, Egypt; 4 Department of Economic Entomology and Pesticides, Faculty of Agriculture, Cairo University, Giza 12613, Egypt; 5 Department of Biology, Faculty of Science, University of Tabuk, Tabuk 71491, Saudi Arabia; ma.alshehri@ut.edu.sa; 6 Department of Biology, College of Science, Princess Nourah bint Abdulrahman University, P.O. Box 84428, Riyadh 11671, Saudi Arabia; laalshuraym@pnu.edu.sa; 7 Nutrition and Food Science, Department of Biology, College of Science, King Khalid University, P.O. Box 960, Abha 61421, Saudi Arabia; hebaghamry882011@gmail.com

References

The original reference number 4 has been retracted and its contents are no longer of reference value. The authors would like to make the following corrections to their published paper [1]:

The authors have decided to replace the previous reference number 4 (El-Far et al.) from the original manuscript with the new reference (Yang et al.) at the same position in the reference list.

Original reference number 4: El-Far, A.H.; Elewa, Y.H.; Abdelfattah, E.-Z.A.; Alsenosy, A.-W.A.; Atta, M.S.; Abou-Zeid, K.M.; Al Jaouni, S.K.; Mousa, S.A.; Noreldin, A.E. Thymoquinone and curcumin defeat aging-associated oxidative alterations induced by D-galactose in rats’ brain and heart. *Int. J. Mol. Sci*. **2021**, *22*, 6839.

New reference number 4: Yang, C.; Wang, S.; Liu, W.; Ma, Z.; Dou, M.; Liu, W.; Li, Q.; Gao, L.; Wang, Y.; Shen, P. Anthocyanidin extract from summer-black-grape affects the expression of Ki-67 in testis, ovary of D-galactose-induced aging mice. *J. Oleo Sci.* **2020**, *69*, 369–376.

The overall numbering and order of the references remain unchanged. The authors state that the scientific conclusions are unaffected. This correction was approved by the Academic Editor. The original publication has also been updated.
