# Supplementary material for: Thymoquinone-Loaded Chitosan Nanoparticles Combat Testicular Aging and Oxidative Stress Through SIRT1/FOXO3a Activation: An In Vivo and In Vitro Study
Source: Pharmaceutics. 2025 Feb 6;17(2):210. doi: 10.3390/pharmaceutics17020210 (PMC11858917; doi:10.3390/pharmaceutics17020210)
Supplement: Supplementary file 1 [file pharmaceutics-17-00210-s001.zip › pharmaceutics-3265167-supplementary.pdf]

**Thymoquinone-Loaded Chitosan Nanoparticles Combat Testicular Aging and Oxidative Stress through SIRT1/FOXO3a Activation -An In Vivo and In Vitro Study**

**Table S1:** Primer Sequences, Accession Numbers, and Product Lengths for Selected Genes

| Gene | Accession Number | Primer Sequences (5'-3') | Product Length (BP) |
|---|---|---|---|
| SIRT1 | NM_001372090.1 | Forward: AAGCAGCTGATACCGTGAAC<br>Reverse: CCGTGTCCATCAATAACGGT | 150 |
| FOXO3 | NM_001106395 | Forward: CCTCTCTGGTGCTTGGACAT<br>Reverse: TGCGTTGACTCTCCTCTTGG | 200 |
| IGF-1 | X06043 | Forward: TGGATGCTCTTCAGTTCGTG<br>Reverse: ACGTCTGGAAGTGGTGCCAT | 180 |
| SOD2 | NM_017051 | Forward: ATGTGGACCTGCCTTACGAC<br>Reverse: GCGTTGATGTGAGGTTCCAG | 130 |
| CAT | NM_012520 | Forward: CAGCCTGAGATGCTTGGATA<br>Reverse: TGGATGTGTAAGGGAAGCCC | 170 |
| PGC-1$\alpha$ | NM_031347 | F: GGTCCCCAGGCAGTAGAT<br>R: CGTGCTCATTGGCTTCATA | 128 |
| PRM1 | NM_001002850.2 | Forward: TGGAGGTGTTGAGAGCCATT<br>Reverse: AGGTCCGTAAGCAGTGAGGT | 190 |
| TERT | NM_053423.1 | Forward: AAGCGTGACATGTGCCCTAT<br>Reverse: CAGGCTCTGGTCTAGGCTGA | 185 |
| GAPDH | NM_ 017008.4 | F: AAAGGGTCATCATCTCCGCC<br>R: AGTGATGGCATGGA CTGTGG- | 197 |

**SIRT1** (Sirtuin 1), **FOXO3** (Forkhead Box O3), **IGF-1** (Insulin-like Growth Factor 1), **SOD2** (Superoxide Dismutase 2), **CAT** (Catalase), **PGC-1$\alpha$** (Peroxisome Proliferator-Activated Receptor Gamma Coactivator 1-alpha), **PRM1** (Protamine 1), and **TERT** (Telomerase Reverse Transcriptase). Additionally, **GAPDH** (Glyceraldehyde 3-Phosphate Dehydrogenase) was used as a reference gene.

**Table S2:** EDX (Elements Composition Data)

| Cs NPs | | | NCP | | |
|---|---|---|---|---|---|
| Formula | mass% | Atom% | Formula | mass% | Atom% |
| C | 26.69 | 35.43 | C | 38.06 | 44.26 |
| N* | 10.96 | 12.48 | N | 17.15 | 17.10 |
| O | 42.41 | 42.27 | O | 43.53 | 37.99 |
| Na | 5.93 | 4.11 | Al | 1.26 | 0.65 |
| K | 14.01 | 5.71 | | | |

**Table S3:** Quantitative Analysis of H9C2 Cell Invasiveness at Different NCP Concentrations

| Concentration | Cell_Count | Relative_Invasion_Index (%) |
|---|---|---|
| Control | 300 | 100.0 |
| Conc 0.01 | 290 | 96.67 |
| Conc 0.1 | 280 | 93.33 |
| Conc 1 | 250 | 83.34 |
| Conc 10 | 200 | 66.66 |
| Conc 100 | 150 | 50.0 |

The relative invasion index is normalized to the control group (set as 100%). This quantitative analysis supports the conclusion that NCP, particularly at a concentration of 100 µg/ml, has an inhibitory effect on H9C2 cell invasiveness.

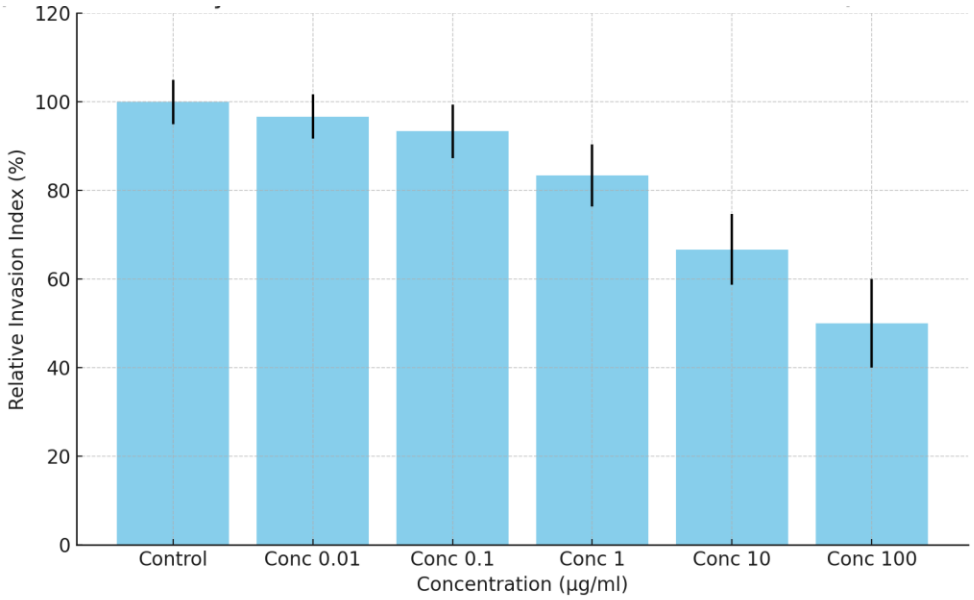


Figure S1: Quantitative analysis of H9C2 cell invasiveness at different NCP concentrations

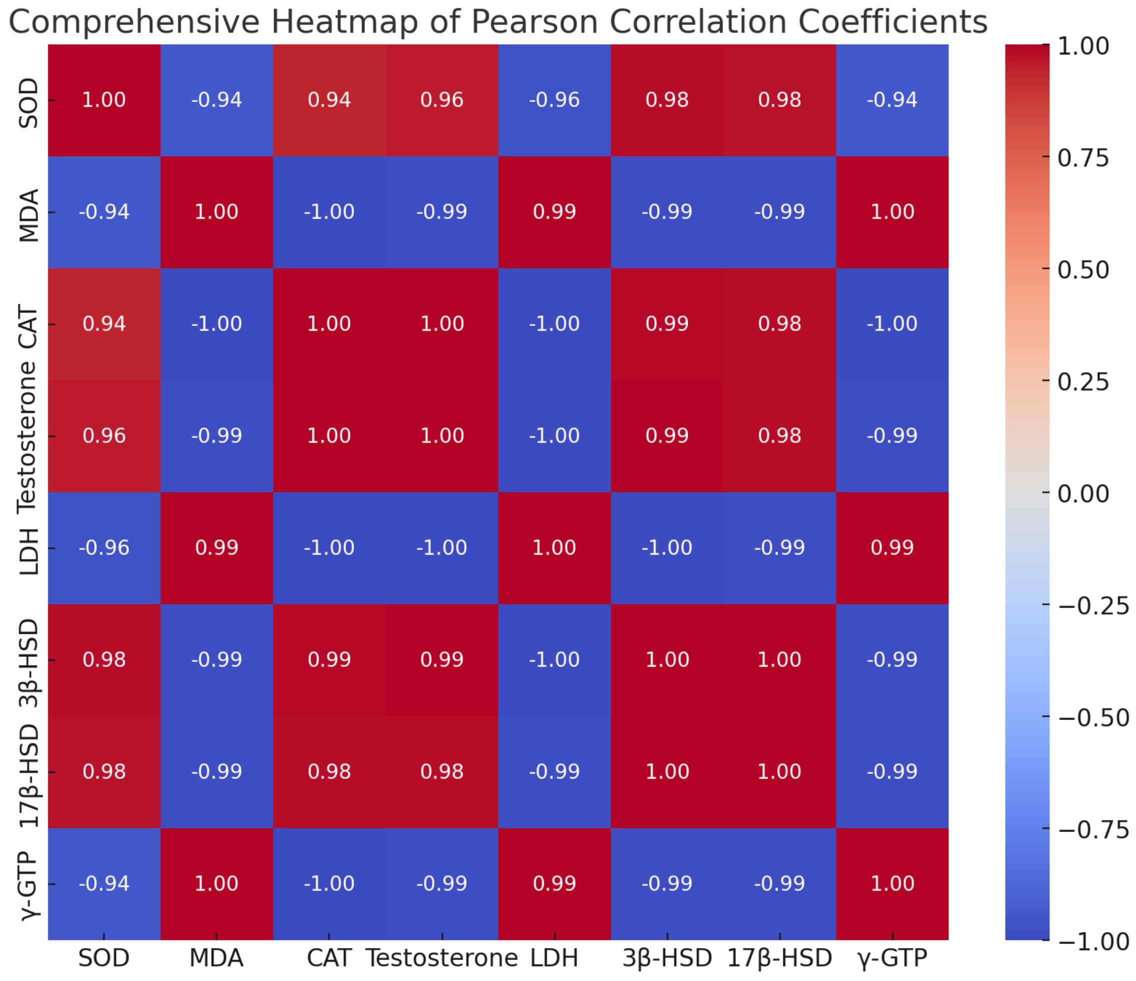


**Figure S2:** Comprehensive Heatmap of Pearson Correlation Coefficients Among Various Biomarkers. Illustrates the Pearson correlation coefficients between different biomarkers measured in the study, including SOD, MDA, CAT, Testosterone, LDH, 3β-HSD, 17β-HSD, and γ-GTP.

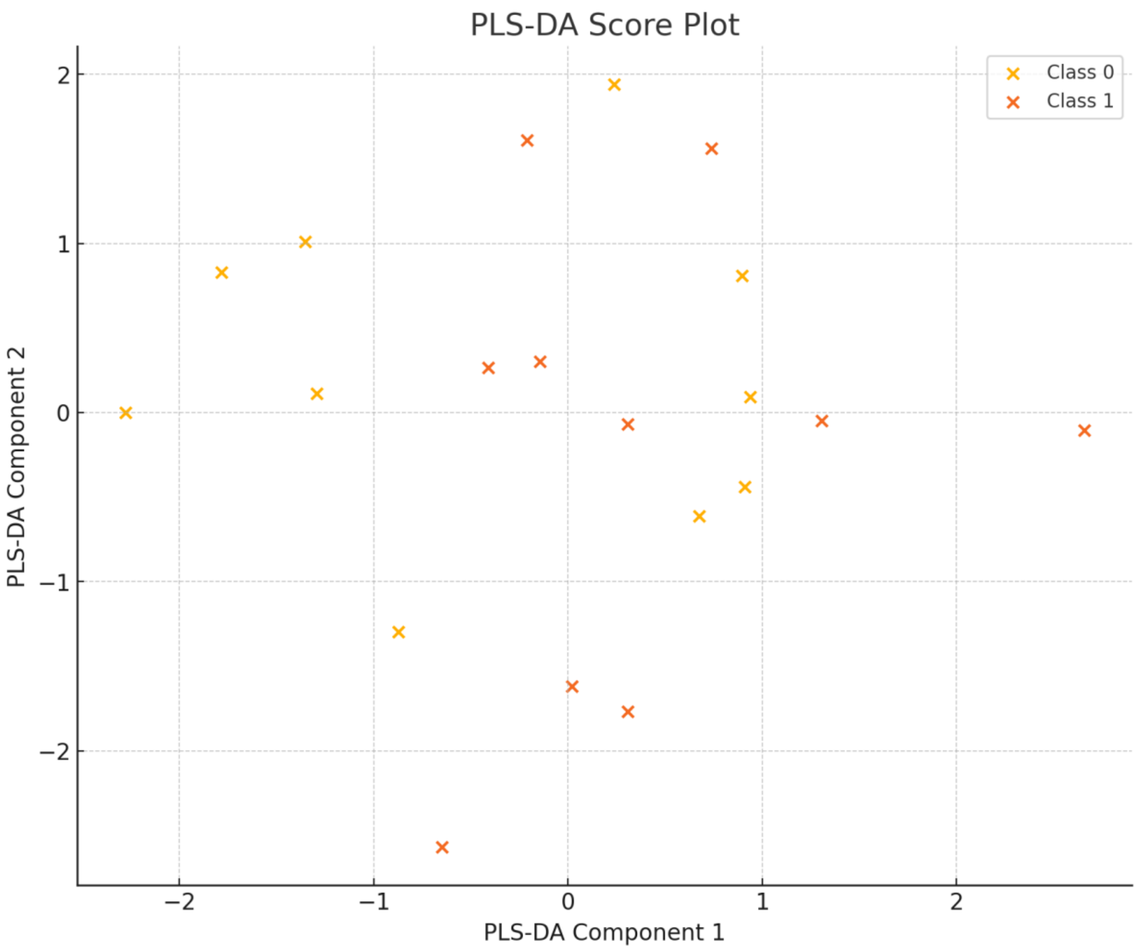


**Figure S3:** The PLS-DA (Partial Least Squares Discriminant Analysis) score plot presented the separation between two different classes (Control and D-gal groups) based on the first two components derived from the PLS-DA model.

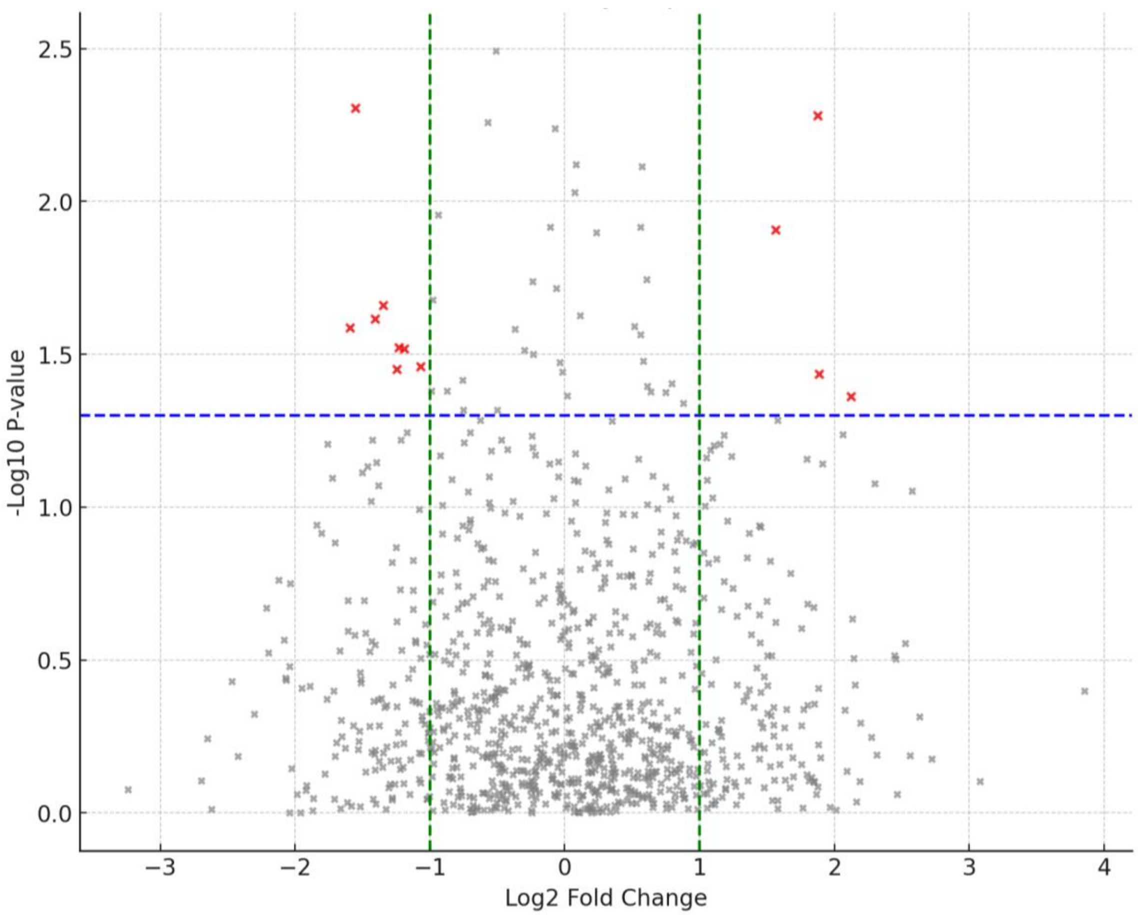


**Figure S4:** Volcano Plot of differentially expressed genes in testis

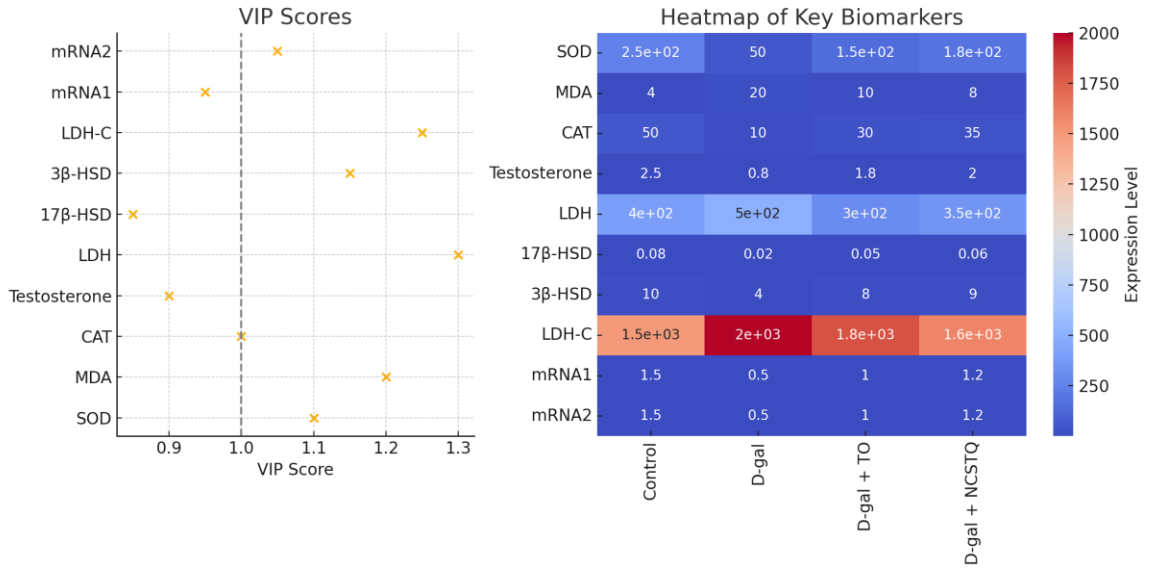


**Figure S5:** VIP Scores and Heatmap of Key Biomarkers.**LDH and LDH-C** have the highest VIP scores (1.3 and 1.25, respectively). This suggests that these biomarkers are critical in distinguishing between the experimental conditions. They could be key indicators of oxidative stress or tissue damage under these conditions.**MDA (Malondialdehyde)** and **SOD (Superoxide Dismutase)** also show relatively high VIP scores (1.2 and 1.1, respectively), indicating their importance in the oxidative stress response. MDA is a marker of lipid peroxidation, while SOD is an antioxidant enzyme, suggesting oxidative damage and defense mechanisms are strongly influenced by the treatments.**17β-HSD and 3β-HSD**, involved in steroid metabolism, also have VIP scores above 1, indicating their role in hormonal regulation in response to the treatments. Biomarkers with slightly lower VIP scores, such as **Testosterone** (0.9) and **CAT (Catalase)** (1.0), are still important but may have a less direct role compared to others. The D-gal-induced model shows signs of oxidative stress, tissue damage, and endocrine disruption. Treatment with **(TQ)** and **NCP** provides varying degrees of protection, with **NCP** showing the most comprehensive recovery in antioxidant defense, tissue integrity, and hormonal balance.
